# Effects of Transcranial Direct Current Stimulation on Motor Function in Children 8–12 Years With Developmental Coordination Disorder: A Randomized Controlled Trial

**DOI:** 10.3389/fnhum.2020.608131

**Published:** 2020-12-11

**Authors:** Melody N. Grohs, Brandon T. Craig, Adam Kirton, Deborah Dewey

**Affiliations:** ^1^Department of Neurosciences, University of Calgary, Calgary, AB, Canada; ^2^Alberta Children's Hospital Research Institute (ACHRI), Calgary, AB, Canada; ^3^Hotchkiss Brain Institute (HBI), University of Calgary, Calgary, AB, Canada; ^4^Department of Pediatrics, University of Calgary, Calgary, AB, Canada; ^5^Department of Community Health Sciences, University of Calgary, Calgary, AB, Canada

**Keywords:** neuromodulation, transcranial direct current stimulation, motor learning, developmental coordination disorder, randomized controlled trial

## Abstract

**Background and objectives:** Developmental coordination disorder (DCD) is a neurodevelopmental motor disorder occurring in 5-6% of school-aged children. It is suggested that children with DCD show deficits in motor learning. Transcranial direct current stimulation (tDCS) enhances motor learning in adults and children but is unstudied in DCD. We aimed to investigate if tDCS, paired with motor skill training, facilitates motor learning in a pediatric sample with DCD.

**Methods:** Twenty-eight children with diagnosed DCD (22 males, mean age: 10.62 ± 1.44 years) were randomized and placed into a treatment or sham group. Anodal tDCS was applied (1 mA, 20 min) in conjunction with fine manual training over 5 consecutive days. Children's motor functioning was assessed with the Purdue Pegboard Test and Jebsen-Taylor Hand Function Test at baseline, post-intervention and 6 weeks following intervention. Group differences in rates of motor learning and skill transfer/retention were examined using linear mixed modeling and repeated measures ANOVAs, respectively.

**Results:** There were no serious adverse events or drop-outs and procedures were well-tolerated. Independent of group, all participants demonstrated improved motor scores over the 5 training days [*F*_(69.280)_, *p* < 0.001, 95% CI (0.152, 0.376)], with no skill decay observed at retention. There was no interaction between intervention group and day [*F*_(2.998)_, *p* = 0.086, 95% CI (−0.020, 0.297)].

**Conclusion:** Children with DCD demonstrate motor learning with long-term retention of acquired skill. Motor cortex tDCS did not enhance motor learning as seen in other populations. Before conclusions of tDCS efficacy can be drawn, additional carefully designed trials with reproducible results are required.

**Clinical Trial Registration:** ClinicalTrials.gov: NCT03453983

## Introduction

Developmental coordination disorder (DCD) affects 5–6% of school-aged children and is characterized by early onset of motor impairment, manifesting as clumsy, slow and inaccurate performance of motor tasks (American Psychiatric Association, 2013). Reduced motor competency interferes with activities of daily living (American Psychiatric Association, 2013), posing a threat to the physical literacy and mental health of affected children (Castelli et al., 2014; Harrowell et al., 2018; Blank et al., 2019). In addition to deficits in motor execution, children with DCD may also display difficulties learning new motor skills and/or tasks (Bo and Lee, 2013; Gomez and Sirigu, 2015; Biotteau et al., 2016a).

Children with DCD are encouraged to practice tasks that they find difficult, in the hope that movement repetition will improve performance (Levac et al., 2009; Smits-Engelsman et al., 2013; Blank et al., 2019). Although successes have be reported with practice, improvements are often variable and more commonly observed with intensive practice (Smits-Engelsman et al., 2013; Jane et al., 2018). The need for high doses of repetition could be attributed to a slower rate of motor learning among children with DCD (Biotteau et al., 2016b; Jane et al., 2018). However, such training may be an anathema to most children, highlighting the need for more efficient therapies.

The use of transcranial direct current stimulation (tDCS), a form of non-invasive brain stimulation, in motor rehabilitation is rapidly expanding (Bikson et al., 2016; Palm et al., 2016). TDCS, through the application of a subthreshold electrical current (1–2 milliamps), alters neuronal excitability and spontaneity, facilitating the brain's endogenous mechanisms of neuroplasticity (Stagg and Nitsche, 2011; Kronberg et al., 2017). When paired with motor skill training, multi-session tDCS is shown to augment motor learning in adults (Reis et al., 2009), typically developing children (Ciechanski and Kirton, 2017; Cole et al., 2018) and children with motor impairment such as cerebral palsy (Finisguerra et al., 2019; Grohs et al., 2019; Saleem et al., 2019). Recent reviews highlight the growing body of research investigating the therapeutic potential of tDCS in children with neurodevelopmental disorders (Finisguerra et al., 2019; Grohs et al., 2019; Saleem et al., 2019); preliminary evidence has supported tDCS enhanced motor functioning in balance, gait, hand function, reaction time and in inhibitory control. Safety and tolerability of tDCS is well-established in adults (Bikson et al., 2016) and is growing in children (Zewdie et al., 2020).

Given support for the safety, feasibility and efficacy of tDCS in pediatric populations with motor impairment, tDCS may provide an avenue to modulate motor learning and strengthen the effects of current therapies in children with DCD. However, the application of tDCS in a pediatric population with DCD has yet to be examined. Here, we present results of the first randomized controlled trial (NCT03453983) investigating the effects of multi-session motor cortex tDCS on motor learning in children with DCD; the primary motor cortex (M1) is a logistical initial target given its direct role in movement production (Sanes and Donoghue, 2000) and evidence showing that plastic changes within M1 are associated with early phases of motor learning (Dayan and Cohen, 2011). Based on previous evidence, it was hypothesized that enhanced rates of motor learning would be observed in children with DCD when fine manual skill training was paired with tDCS.

## Materials and Methods

### Enrollment

This study was carried out between July 2018 and November 2019 at the Alberta Children's Hospital, Calgary, Canada. Participants were recruited through developmental and community pediatricians, psychologists, physical/occupational therapists and via social media. Written informed consent from participants' legal guardians and child assent were obtained at enrollment. The University of Calgary Conjoint Health Research Ethics Board approved this study (REB18-0183).

### Eligibility

Inclusion criteria were: (1) age 8 to 12 years, (2) current diagnoses of DCD by a registered health care provider and (3) right-handed (i.e., hand used for writing). Children 8–12 years were recruited as DCD is commonly diagnosed in elementary school. Those with pre-term birth (<36 weeks' gestation) or any neuropsychiatric, neurological and/or chronic disorders were excluded. Children with a diagnosis of attention deficit/hyperactivity disorder (ADHD), learning disorder (LD), or generalized anxiety disorder (GAD) were included given the high co-occurrence with DCD (Dewey et al., 2002; Dewey, 2018).

Participants were screened to ensure they met clinical criteria for DCD outlined in the Diagnostic and Statistical Manual of Mental Disorders (DSM-5) (American Psychiatric Association, 2013). Children demonstrated motor deficits (criterion A) with Total Test scores below the 16th percentile on the Movement Assessment Battery for Children 2nd Edition (MABC-II) (Barnett et al., 2007). Motor deficits interfered with daily functioning (criterion B), began early in development (criterion C), and were not better explained by an intellectual disability, visual impairment or neurological condition (criterion D). Diagnostic criteria B and C were confirmed by a parent questionnaire developed by the investigators, which included questions about difficulties experienced in three domains, (1) motor (i.e., handwriting, riding a bike, self-care tasks, motor planning, learning new motor tasks, etc.), (2) social (play and social skills, physically tired, lack of energy, etc.), and (3) academic (reading, writing, math skills, etc.), as well as the age at which motor difficulties were first observed. Criterion D was confirmed by questions on the parent questionnaire regarding all prior and current diagnoses as well as visual impairments, and children obtaining a Full-Scale IQ score >79 on the Wechsler Abbreviated Scale of Intelligence 2nd Edition (WASI-II) (Wechsler, 2011).

### Study Design

A randomized, double-blind, sham-controlled trial was conducted in accordance with CONSORT guidelines (Schulz et al., 2010), including pediatric-specific considerations (Saint-Raymond et al., 2010). After screening, children were randomly assigned without stratification to one of two parallel intervention groups: (1) active tDCS or (2) sham. A simple randomization procedure was used. Allocation (1:1) was concealed in sequentially numbered, opaque, sealed envelopes. Corresponding envelopes were opened by the investigator (MNG) immediately before intervention. Participants were blinded to their assigned group throughout the study and completed a post-intervention questionnaire that asked them to guess which intervention they received and why. Investigators were blinded at data analysis.

### Sample Size

Previous evidence of tDCS enhanced motor learning in typically developing children and adolescents, using a similar protocol, reported a moderate-to-large effect size (Cohens *d* > 0.65) (Cole et al., 2018). To estimate the sample size required for the primary linear mixed model analysis in the current study, the smpsize_lmm command in RStudio was used with the above effect size and a two-sided type 1 error of 0.05. It was estimated that 16 participants per group would have 90% power to detect group differences in the primary outcome measure.

### tDCS Intervention

The right M1 was localized using the 10–20 electroencephalography method (Steinmetz et al., 1989). A saline soaked 25cm^2^ sponge electrode was placed over the right M1 (active anode electrode), with a second identical electrode placed on the contralateral supraorbital region (reference cathode electrode), held in place by adjustable head straps consistent with established methods (Ciechanski and Kirton, 2017).

Electrodes were attached to a conventional 1x1 tDCS system (Soterix, NY). Current was ramped up to 1 mA over 30 seconds (s). After 120 s, the current was maintained for 20 min (active tDCS group) or ramped back down to 0 mA over 30 s (sham group). The initial ramp-up produces transient scalp sensations and has been established as a valid sham technique (Ambrus et al., 2012). Following each stimulation session, participants completed a safety, and tolerability questionnaire (Garvey et al., 2001), documenting symptoms (i.e., headaches, burning, itching, tingling, and nausea), their severity and duration, as well as tolerability compared to seven common childhood experiences.

### Outcome Measures

The primary outcome was left hand Purdue Pegboard Test (PPT) performance, a validated assessment of fine motor coordination and hand dexterity (Tiffin and Asher, 1948). The PPT consists of four subtests: left hand [PPT_L_], right hand [PPT_R_], bimanual [PPT_LR_] peg placement, and bimanual assembly [PPT_A_]. The peg placement tasks involved placing as many pins as possible into a pegboard in 30 s. The assembly task involved building as many copies of a demonstration structure using pins, collars and washers in 60 s. Scores were the highest total number of placed pegs or assembled items. The PPT_L_ (non-dominant left-hand performance) was used for motor skill training and as the primary outcome measure of motor learning, as it is a challenging fine manual task for children to learn without reaching a learning “ceiling” effect.

Motor skills may be acquired by two modes of learning: online and offline. Online learning refers to skill learning that occurs within a training period. Offline learning refers to skill learning that occurs after the training session has ended and is often referred to as consolidation. TDCS may differentially affect online and offline learning (Reis et al., 2009). In the current study, online effects (within-day training) were determined by comparing baseline to final PPT_L_ scores each day. Offline effects (between-day consolidation) were quantified by comparing baseline PPT_L_ scores each day to final PPT_L_ scores from the previous day. Daily effects were summed to obtain total online and offline effects.

Secondary outcomes included PPT_R_, PPT_LR_, and PPT_A_ performance, to examine intervention effects on the untrained hand and bimanual skills, as well as Jebsen-Taylor Test of Hand Function (JTT) performance (Jebsen, 1969), an upper extremity test of unimanual motor skills. The JTT included five subtests: card turning, picking up/placing small objects, stacking checkers, moving light objects, and moving heavy objects. Left and right hands were tested independently. An overall score for each hand was obtained by summing the completion times for each subtest [JTT_R_, JTT_L_].

### Motor Training

**Figure 1:**
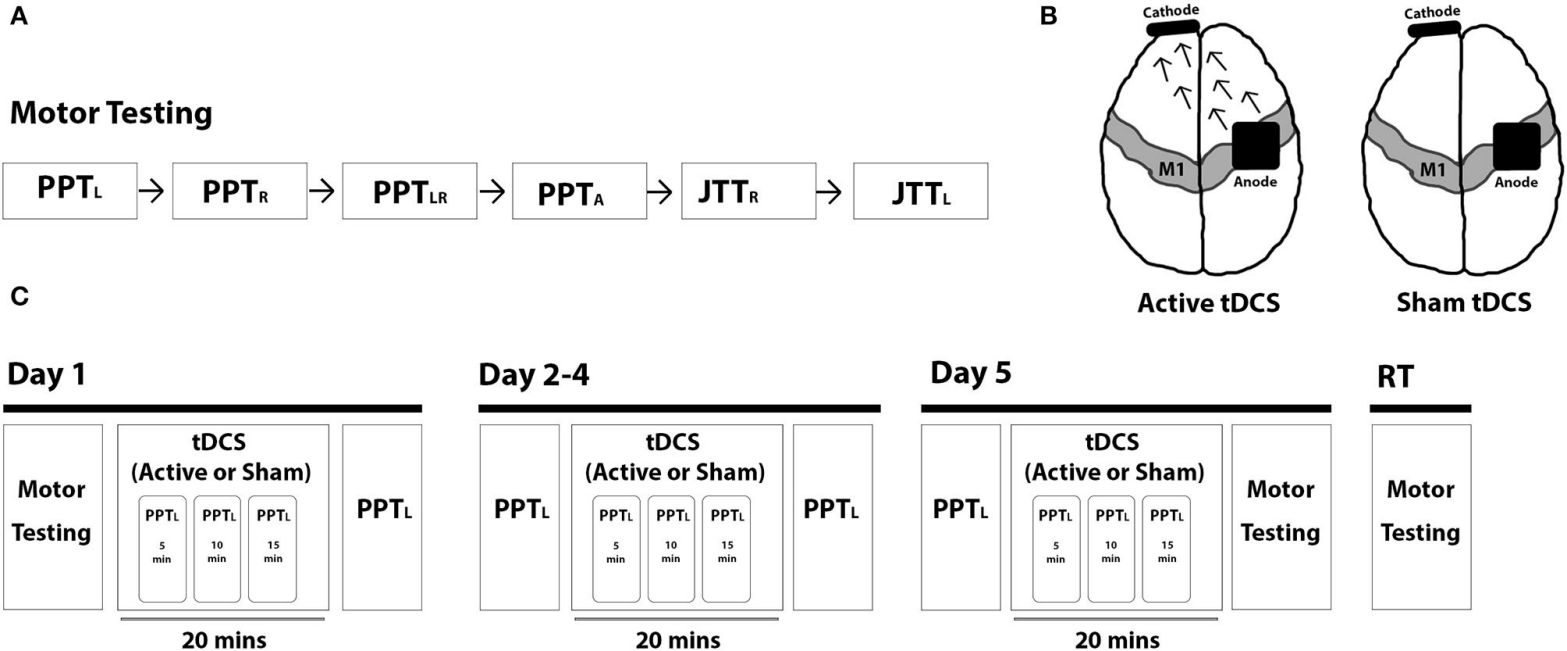
Trial protocol. **(A)** Motor skill testing included the Purdue Pegboard Test (PPT) (left-handed: PPT_L_, right-handed: PPT_R_, bimanual: PPT_LR_, assembly: PPT_A_) and the Jebsen-Taylor Test of Hand Function (JTT) (right-handed: JTT_R_, left-handed: JJT_L_). **(B)** Intervention groups included 1 mA anodal tDCS (left; arrows represent the direction of current flow from anode to cathode) and sham tDCS (right). **(C)** Study protocol is shown broken down by each intervention day (day 1–5) and for retention testing (RT) at 6-weeks post-intervention.

A schematic of the study protocol is shown in Figure 1. On day 1, baseline motor tests (PPT, JTT) were administered, followed by tDCS intervention (active or sham). During the intervention, three PPT_L_ trials were performed at 5, 10, and 15 min as well as after intervention. Participants repeated this protocol for 4 consecutive days (days 2–5). Following training on day 5, all motor tests were repeated. Participants returned 6 ± 1 weeks later to repeat all motor tests. Assessments were video recorded and blindly scored offline.

### Analysis

Statistical analysis was performed in RStudio (RStudio Team, V1.3.1093) (R Core Team, 2017) and SPSS (IBM SPSS Software, V25) (SPSS Inc., 2017). Shapiro-Wilk tests assessed normality of each measure. As appropriate, independent samples t-tests, Mann-Whitney *U*-tests or chi-square tests compared participant characteristics, clinical and motor scores at baseline and tolerability ratings between intervention groups. The primary analysis was intention-to-treat and involved all participants.

Our statistical approach was based on previously established methods (Cole et al., 2018). A linear mixed effects model was chosen for the primary analysis as this approach offers advantages for longitudinal data sets with more data points and non-linear outcomes (Gibbons et al., 2010); our primary outcome parameter (change in PPT_L_ score) was measured at six timepoints and previous findings from studies using similar protocols (Ciechanski and Kirton, 2017; Cole et al., 2018) showed non-linear changes in PPT_L_ performance over multiple training days. The linear mixed effects model examined changes in the primary outcome (PPT_L_) between groups from pre- to post-intervention with fixed effects for Group, Day, the interaction of Group and Day, and random effects for participants including the intercept to account for repeated measures.

As secondary motor outcomes were measured at fewer timepoints and motor learning curves were not being generated, two-way repeated measures ANOVAs were used to investigate changes in secondary outcomes between intervention groups. Independent samples *t*-tests examined between group differences in performance at each time point. Paired samples t-tests examined within group differences in PPT (PPT_L_, PPT_R_, PPT_LR_, and PPT_A_) and JTT (JTT_L_, JTT_R_) performance between timepoints (i.e., baseline to post-intervention, baseline to retention) and potential skill decay between the final training block (day 5) and retention testing. Online and offline learning effects were explored within and between groups using paired and independent samples t-tests.

## Results

### Population

**Figure 2:**
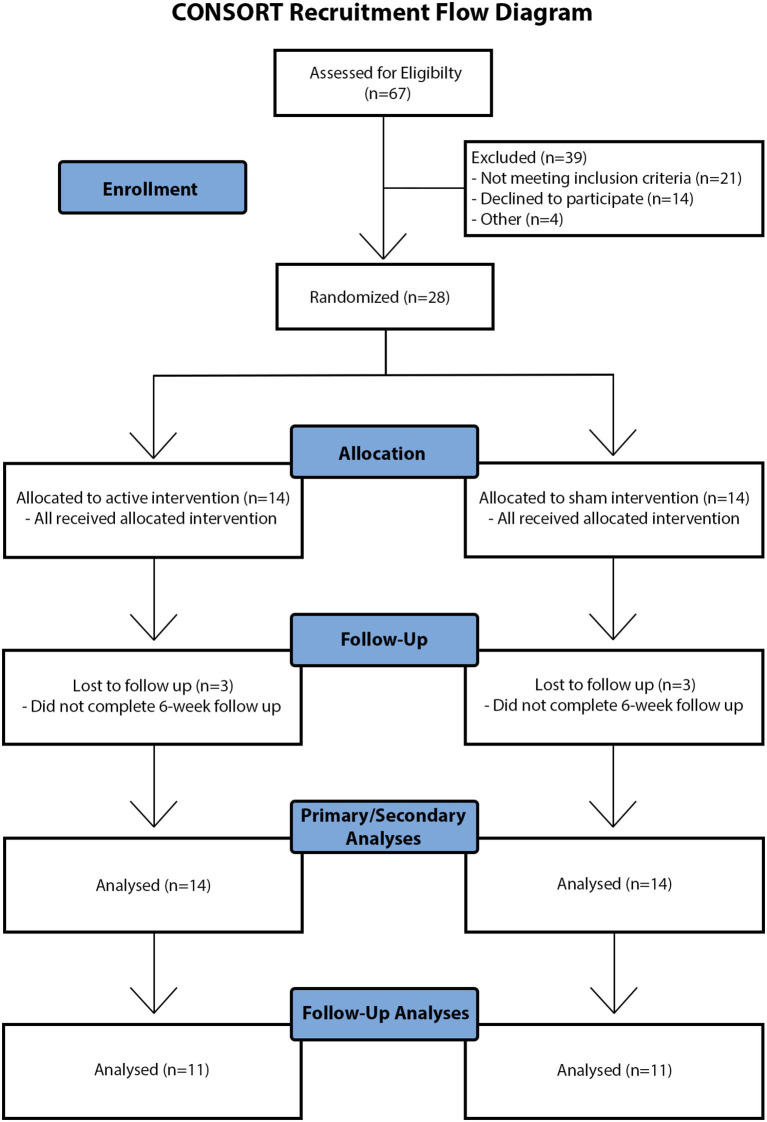
CONSORT recruitment flow diagram. Visual schematic of participant recruitment, screening, data collection, and analysis. Note that “other reasons” for exclusion of children at eligibility screening and follow-up included travel and family factors.

Twenty-eight children with DCD [10.62 ± 1.44 years; 22 (79%) male] were randomized (14 active, 14 sham). All participants completed baseline motor skill testing, the 5 consecutive intervention days and post-intervention motor skill testing. Six participants (3 active, 3 sham) did not complete retention motor skill testing due to travel or family factors (Figure 2, CONSORT recruitment flow diagram). Group demographics, clinical scores and baseline motor scores are shown in Table 1. No group differences were observed for age [*t*_(26)_ = 0.637, *p* = 0.530], sex [*x*^2^(1) = 0.848, *p* = 0.357] or clinical scores (MABC-II: *U* = 79, *p* = 0.374; WASI-II: *t*_(26)_ = −0.586, *p* = 0.563). No group differences in baseline motor scores were observed (all *p* > 0.7). Fifteen participants had ADHD [*n* = 6 (43%) active, *n* = 9 (64%) sham], 11 had a LD [*n* = 6 (43%) active, *n* = 5 (36%) sham], and 5 had GAD [*n* = 3 (21%) active, *n* = 2 (14%) sham] (Table 2). Proportions did not differ between groups (all *p* > 0.2). Thirteen of the 28 participants were taking medications for ADHD (i.e., Vyvanse, Biphentin, and Clonidine) and/or anxiety (i.e., Prozac, Zoloft, and Citalopram) (Table 2). Proportions did not differ between groups (*p* > 0.7).

**Table 1:** Participant demographics, clinical scores and baseline motor scores by intervention group.

**Group**	**Age (years)**	**Sex (M:F)**	**WASI-II FSIQ**	**MABC-II total test**	**Baseline Purdue Pegboard Test (PPT) scores**				**Baseline Jebsen-Taylor Test (JTT) scores**	
					**PPT_**L**_**	**PPT_**R**_**	**PPT_**LR**_**	**PPT_**A**_**	**JTT_**L**_**	**JTT_**R**_**
Active (*n* = 14)	10.80 (±1.42)	10:4	100.29 (±9.88)	3.5 (±0.51)	11.52 (±2.10)	12.05 (±1.93)	8.93 (±1.67)	20.14 (±4.77)	29.48 (±5.16)	27.78 (±3.97)
Sham (*n* = 14)	10.45 (±1.50)	12:2	102.93 (±3.66)	2.86 (±0.38)	11.77 (±2.79)	12.26 (±2.42)	8.98 (±2.25)	20.50 (±6.49)	29.19 (±4.24)	27.68 (±4.98)
Mean	10.62 (±1.44)	22:6	101.61 (±2.23)	3.18 (±0.32)	11.64 (±2.43)	12.15 (±2.15)	8.96 (±1.95)	20.32 (±5.59)	29.33 (±4.64)	27.73 (±4.42)
Between group (*p*-value)	0.530	0.357	0.563	0.374	0.791	0.798	0.950	0.869	0.871	0.953

*Note: Values are shown as group means ± standard deviation. Test statistics are shown for between group and within group comparisons*.

*M:F, ratio of males to females; WASI-II, Wechsler Abbreviated Scale of Intelligence 2nd Edition; MABC-II, Movement Assessment Battery for Children 2nd Edition; PPT_L_, Purdue Pegboard Test left-hand; PPT_R_, Purdue Pegboard Test right-hand; PPT_LR_, Purdue Pegboard Test bimanual; PPT_A_, Purdue Pegboard Test assembly; JTT_L_, Jebsen-Taylor Test left-hand; JTT_R_, Jebsen Taylor Test right-hand*.

**Table 2:** Distribution of co-occurring attention, learning and anxiety disorders by participant and group.

**Participant (DCD)**	**Attention disorder**	**Learning disorder**	**Anxiety disorder**	**Prescribed medications**
**Active tDCS Group**				
S1	–	X	–	–
S2	–	–	X	–
S3	X	–	–	–
S4	X	X	X	X
S5	X	–	–	X
S6	–	–	–	–
S7	X	X	–	X
S8	–	X	–	X
S9	–	X	–	X
S10	X	–	–	X
S11	–	–	–	–
S12	X	X	X	–
S13	–	–	–	–
S14	–	–	–	–
Active total	6	6	3	6
**Sham tDCS Group**				
S1	X	–	–	X
S2	–	X	–	–
S3	X	–	–	X
S4	–	–	–	X
S5	X	X	–	–
S6	X	–	–	–
S7	–	–	–	–
S8	X	–	–	X
S9	X	–	–	X
S10	X	X	X	X
S11	X	X	X	X
S12	–	–	–	–
S13	X	X	–	–
S14	–	–	–	–
Sham total	9	5	2	7
Between group (*p*-value)	0.256	0.699	0.622	0.705

*Note: The presence of a diagnosis by a registered health care provider is denoted with an X. Total within group numbers and test statistics for between group differences in distribution are also shown*.

### Motor Learning

**Figure 3:**
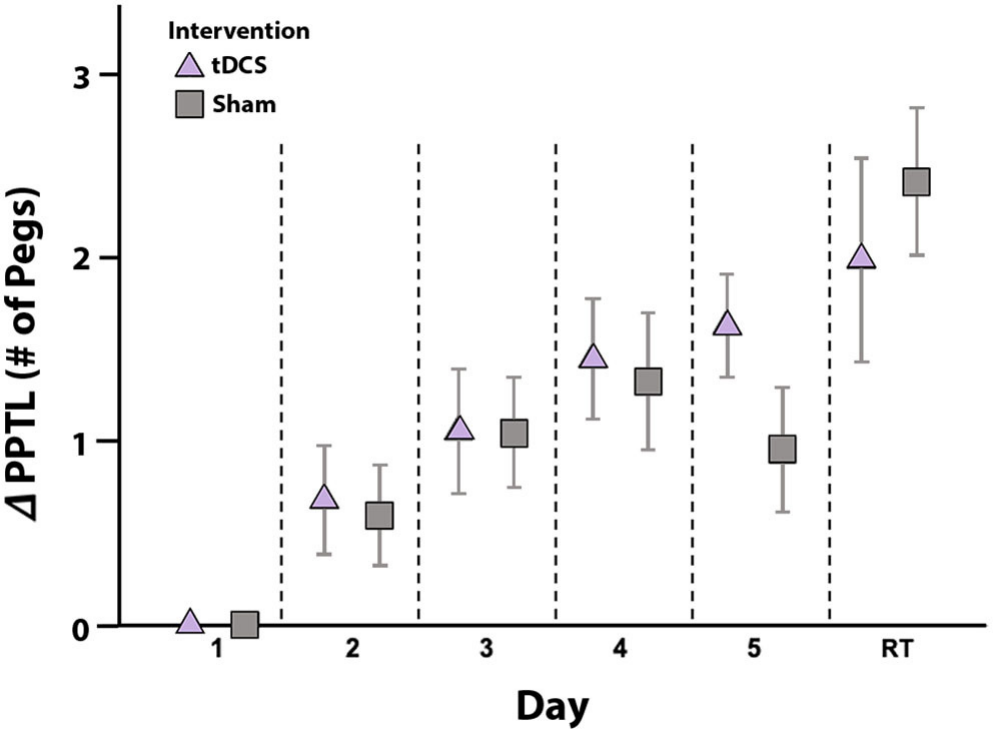
Motor learning by intervention group. Mean daily change in PPT_L_ scores from baseline (y-axis) are shown for the active (triangle) and sham (squares) intervention groups. Error bars represent standard error. In both groups, scores improved from baseline to post-intervention testing on day 5, with no skill decay at retention testing (RT) 6-weeks post-intervention. No between group differences in PPT_L_ scores were noted at any timepoint. ΔPPTL: change in left-hand Purdue Pegboard Test scores from baseline.

PPT_L_ learning curves by group are shown in Figure 3. Curves were generated by plotting mean change in score from baseline to each training point. Linear mixed effects modeling showed that, independent of intervention, all participants demonstrated motor learning over 5 training days [*p* < 0.001, 95% CI (0.25–0.41), Table 3]. No interaction effect of Day and Group on rate of motor learning was seen; therefore, the interaction term was removed from the final mixed model [PPT_L_ ~ group + day + (1|subjects)]. Average PPT_L_ performance was higher on post-intervention day 5 compared to baseline in both groups [active: *t*_(13)_ = −5.824, *p* < 0.001, Cohen's *d* = 1.557; sham: *t*_(13)_ = −2.820, *p* = 0.014, Cohen's *d* = 0.754]. No group differences were observed in average PPT_L_ performance at any time point (all *p* > 0.1).

**Table 3:** Results of linear mixed effects model examining motor learning over 5 days of skill training.

	**PPT**_****L****_ **score**		
	**Estimates**	**CI**	***P***
**Fixed effects**			
Intercept	11.41	8.91–13.90	<0.001^*^
Group	0.07	−1.50–1.64	0.927
Day	0.33	0.25–0.41	<0.001^*^
**Random effects**			
Subjects	4.41	-	-
ICC	0.90	-	-
Marginal *R*^2^	0.044	-	-

* *Bold values represent statistically significant findings with a p < 0.001. CI, Confidence interval*.

### Retention

Learning effects were retained in both groups, with no skill decay in PPT_L_ scores between post-intervention day 5 and retention testing at 6-weeks (Figure 3). In the active group, PPT_L_ scores at retention did not differ from post-intervention day 5 [*t*_(10)_ = −1.966, *p* = 0.078, Cohen's *d* = 0.593]. Within the sham group, higher PPT_L_ scores were observed at retention compared to post-intervention day 5 [*t*_(10)_ = −4.989, *p* = 0.001, Cohen's *d* = 1.504]. This difference may relate to lower scores in the sham group on day 5 (see above). In both groups, PPT_L_ scores at retention were higher than baseline [active: *t*_(10)_ = −3.585, *p* = 0.005, Cohen's *d* = 1.080; sham: *t*_(10)_ = −6.037, *p* < 0.001, Cohen's *d* = 1.820], with no group differences [*t*_(20)_ = −1.025, *p* = 0.321].

### Online and Offline Learning Effects

**Figure 4:**
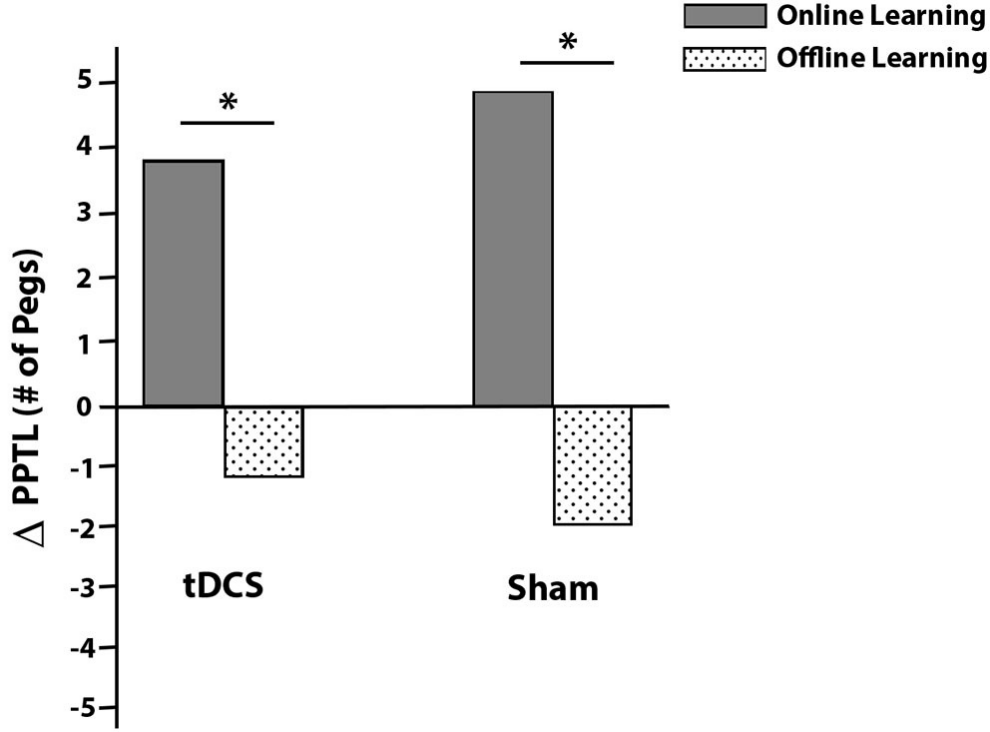
Average PPT_L_ online (solid gray) and offline (dotted) learning effects by intervention group. Online effects are within session improvements while offline effects are improvements that occur between sessions (consolidation). Daily online and offline effects were summed to obtain total online and offline changes in scores (y-axis ΔPPTL). *p < 0.05.

There was more online learning compared to offline learning in both the active [*t*_(13)_ = 2.545, *p* = 0.024] and sham [*t*_(13)_ = 5.488, *p* < 0.001] groups (Figure 4). No group differences in online [*t*_(26)_ = −0.669, *p* = 0.509] or offline [*t*_(26)_ = 0.866, *p* = 0.395] learning were observed.

### Secondary Motor Outcomes

**Figure 5:**
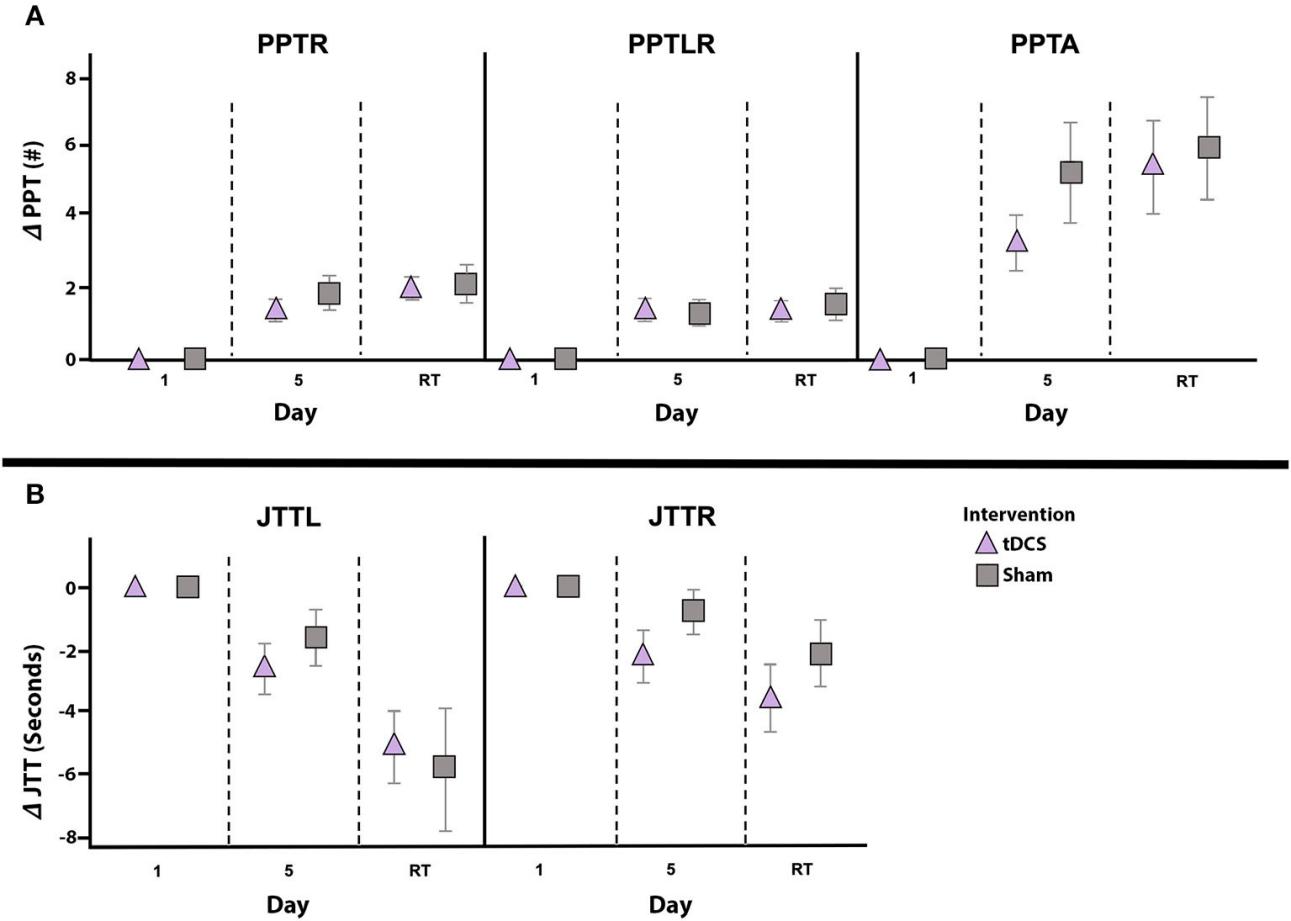
Change in secondary outcomes measures by intervention group. Mean daily score change from baseline (y-axes) for PPT_R_, PPT_LR_, and PPT_A_ **(A)** as well as JTT_L_ and JTT_R_ **(B)**, shown for the active (triangle) and sham (squares) groups. Error bars represent standard error. Independent of intervention group all scores significantly improved from baseline (day 1) to post-intervention testing on day 5, and from baseline to retention testing (RT) 6-weeks post-intervention. No between group differences in performance on any secondary measures were noted at any timepoint. Note that negative JTT scores indicate improved performance (i.e., reduction in completion time) and positive JTT scores indicate worse performance (i.e., increased time required to complete tasks). PPT_L_, Purdue Pegboard Test left-hand; PPT_R_, Purdue Pegboard Test right-hand; PPT_LR_, Purdue Pegboard Test bimanual; PPT_A_, Purdue Pegboard Test assembly; JTT_L_, Jebsen-Taylor Test left-hand; JTT_R_, Jebsen-Taylor Test right-hand.

Effects of intervention on secondary untrained PPT and JTT measures are shown in Figure 5. Learning effects were observed for PPT_R_ (*F* = 32.346, *p* < 0.001, partial eta^2^ = 0.554), PPT_LR_ (*F* = 32.795, *p* < 0.001, partial eta^2^ = 0.558) and PPT_A_ (*F* = 28.041, *p* < 0.001, partial eta^2^ = 0.519). These were independent of group, with no interaction effects of Time and Group (all *p* > 0.2). In both groups, compared to baseline, PPT_R_ scores were higher on post-intervention day 5 [active: *t*_(13)_ = −4.535, *p* = 0.001, Cohen's *d* = 1.212; sham: *t*_(13)_ = −3.863, *p* = 0.002, Cohen's *d* = 1.032] and at retention [active: *t*_(10)_ = −6.297, *p* < 0.001, Cohen's *d* = 1.899; sham: *t*_(10)_ = −4.856, *p* = 0.001, Cohen's *d* = 1.464]. PPT_LR_ scores were higher by day 5 [active: *t*_(13)_ = 4.436, *p* = 0.001, Cohen's *d* = 1.186; sham: *t*_(13)_ = 3.721, *p* = 0.003, Cohen's *d* = 0.994] and at retention [active: *t*_(10)_ = −4.730, *p* = 0.001, Cohen's *d* = 1.426; sham: *t*_(10)_ = −4.351, *p* = 0.001, Cohen's *d* = 1.312] for both groups. PPT_A_ scores demonstrated a similar pattern at day 5 [active: *t*_(13)_ = −4.200, *p* = 0.001, Cohen's *d* = 1.122; sham: *t*_(13)_ = −3.727, *p* = 0.003, Cohen's *d* = 0.996] and retention [active: *t*_(10)_ = −4.139, *p* = 0.002, Cohen's *d* = 1.248; sham: *t*_(10)_ = −4.967, *p* = 0.001, Cohen's *d* = 1.498]. Finally, no skill decay from post-intervention day 5 to retention testing was observed in either group for PPT_R_ [active: *t*_(10)_ = −2.015, *p* = 0.072, Cohen's *d* = 0.608; sham: *t*_(10)_ = −0.586, *p* = 0.571, Cohen's *d* = 0.177], PPT_LR_ [active: *t*_(10)_ = 0.379, *p* = 0.712, Cohen's *d* = 0.114; sham: *t*_(10)_ = −2.036, *p* = 0.069, Cohen's *d* = 0.614], or PPT_A_ [active: *t*_(10)_ = −1.919, *p* = 0.084, Cohen's *d* = 0.579; sham: *t*_(10)_ = −0.576, *p* = 0.578, Cohen's *d* = 0.174].

Independent of intervention, learning effects were observed for JTT_L_ (*F* = 11.476, *p* = 0.002, partial eta^2^ = 0.306) and JTT_R_ (*F* = 7.887, *p* = 0.009, partial eta^2^ = 0.233). In the active group, JTT_L_ and JTT_R_ performance was faster on post-intervention day 5 [JTT_L_ *t*_(13)_ = 3.150, *p* = 0.008, Cohen's *d* = 0.842; JTT_R_ *t*_(13)_ = 2.700, *p* = 0.018, Cohen's *d* = 0.722] and at retention [JTT_L_ *t*_(10)_ = 4.397, *p* = 0.001, Cohen's *d* = 1.326; JTT_R_ *t*_(10)_ = 3.348, *p* = 0.007, Cohen's *d* = 1.009] compared to baseline. In the sham group, improved JTT_L_ performance was not seen on day 5 [*t*_(13)_ = 1.722, *p* = 0.109] but was present at retention testing [*t*_(9)_ = 3.769, *p* = 0.004, Cohen's *d* = 1.136]. JTT_R_ scores at day 5 [*t*_(13)_ = 1.164, *p* = 0.265, Cohen's *d* = 0.311] and retention [*t*_(9)_ = 2.033, *p* = 0.073, Cohen's *d* = 0.613] did not differ from baseline in the sham group. There was no evidence of skill decay from day 5 to retention testing on the JTT_L_ [active: *t*_(10)_ = 2.093, *p* = 0.063, Cohen's *d* = 0.631; sham: *t*_(9)_ = 1.589, *p* = 0.147, Cohen's *d* = 0.479] or JTT_R_ [active: *t*_(10)_ = 1.521, *p* = 0.159, Cohen's *d* = 0.459; sham: *t*_(9)_ = 0.301, *p* = 0.770, Cohen's *d* = 0.091].

### Safety, Tolerability, and Blinding

In total, 140 tDCS sessions were performed with no serious adverse events and sessions were well-tolerated. Reported sensations included itching (89%; 44% mild, 48% moderate, 8% severe), tingling (68%; 79% mild, 5% moderate, 16% severe), and burning (54%; 73% mild, 27% moderate), which did not differ by group. Seven participants reported a mild headache lasting for the first few minutes of stimulation and five participants felt mildly nauseated in a single session. TDCS tolerability rankings, on an 8-point scale, were similar for the active (4.1 ± 1.1) and sham groups (4.1 ± 1.2; p = 0.974) and were comparable to watching TV (2.6 ± 0.9) or a long car ride (5.1 ± 1.3). Participants were unable to predict their treatment group (44% accuracy, 50% indicates chance).

## Discussion

The current trial is the first to examine the therapeutic efficacy of tDCS on motor learning in children with DCD. Independent of intervention, all children's motor performance improved over the 5 training days and skill improvements were retained for 6 weeks. Contrary to our hypothesis, excitatory stimulation of the right primary motor cortex did not enhance motor learning.

The research literature suggests that poor motor performance in children with DCD may be associated with deficits in motor learning (Bo and Lee, 2013; Biotteau et al., 2016a). However, research concerning the presence of motor learning deficits in DCD is inconsistent, with some studies reporting limited skill improvement following practice (Kagerer et al., 2004; Gheysen et al., 2011; Zwicker et al., 2011) and others reporting positive effects of practice (Ferguson et al., 2013; Lejeune et al., 2013; Mombarg et al., 2013; Smits-Engelsman et al., 2015). Studies supporting the latter emphasize that children with DCD are able to acquire motor skills, though they may display slower rates of motor learning, requiring more intensive practice to reach desired levels of motor competence. In the current trial, fine motor performance of the non-dominant limb improved significantly with practice, independent of intervention. This finding supports the capacity of children with DCD to learn novel motor skills.

Motor learning involves both online and offline processes. Online learning includes skill gains obtained during active training, whereas offline learning includes gains occurring between training sessions (i.e., consolidation). Within both groups, the majority of motor learning took place online. This suggests that children with DCD may show less efficient offline motor learning, or consolidation, which has been previously suggested in the DCD literature (Zwicker et al., 2011) and warrants further study.

Motor skill retention and transfer to untrained tasks are also features of successful motor learning (Muratori et al., 2013). We show no evidence of skill decay in either group between the final training day and retention testing at 6-weeks. Moreover, motor skill improvements were not restricted to the trained hand or task as improvements on all secondary motor outcomes were observed. Learning effects were generalized to the untrained dominant hand. Taken together, these results suggest that children with DCD display intact motor skill acquisition, adaptation, retention, and transfer following practice.

Contrary to previous evidence of tDCS enhanced motor learning in typically developing children (Ciechanski and Kirton, 2017; Cole et al., 2018) and children with motor impairment (i.e., cerebral palsy) (Grohs et al., 2019), tDCS did not enhance the rate of motor learning in children with DCD relative to practice alone. The limited efficacy of tDCS could be reflective of stimulation parameters including cortical target (Thibaut et al., 2017) or montage (i.e., anode and cathode arrangement) (Woods and Martin, 2016). Although M1 is a common target to modulate motor learning due its direct role in motor production (Todorov, 2003), other structures may be better suited to the DCD population. For instance, dysfunction in cerebellar networks has commonly been identified in DCD (Biotteau et al., 2016a). Given the role of the cerebellum in motor control and learning (Manto et al., 2012), as well as positive findings from trials implementing cerebellar tDCS for motor impairment (Celnik, 2015), it may be a promising target in DCD.

Regarding montage, different anode/cathode placement uniquely modulates cortical excitability. Anodal tDCS involves placement of the anode over a target region and generally produces excitatory effects within the cortex, whereas in cathodal tDCS the cathode is placed over the target region producing an overall inhibitory effect. Although anodal stimulation was chosen here based on previous evidence (Cole et al., 2018), cathodal stimulation has also been shown to enhance motor learning in children (Ciechanski and Kirton, 2017). Neurophysiological research has reported reduced interhemispheric inhibition of M1 activity in DCD (He et al., 2018). It is, therefore, possible that inhibiting cortical activity via cathodal stimulation may produce favorable outcomes in children with DCD. Future studies that characterize baseline cortical excitability and neurometabolites, using techniques such as transcranial magnetic stimulation (TMS) and magnetic resonance spectroscopy (MRS), could help in refining application (i.e., stimulation intensity, montage, and target).

It is also possible that there are no effects of tDCS in children with DCD. However, given this is the first study to examine effects of neuromodulation in children with DCD, future studies using well-supported protocols that target different cortical regions and/or examine different montages are highly encouraged. Finally, given that tDCS enhances motor learning via facilitating endogenous neuroplastic mechanisms, the absence of response to tDCS observed here could also suggest disordered neuroplastic mechanisms in individuals with DCD. Future studies utilizing techniques such as TMS could help to elucidate plasticity mechanisms in DCD.

### Limitations

Our sample size calculation estimated that 16 participants per group would provide us with 90% power to detect group differences; however, our final groups consisted of 14 participants. As a result, our sample size may have decreased our ability to detect potential group differences, or efficacy, and may have limited the generalizability of our findings. There was also a high degree of variability in performance on our outcome measures, which may have decreased our ability to detect group differences given the sample size. Thirteen participants were on medications that influence neurotransmitter systems and could have impacted tDCS efficacy (McLaren et al., 2018). Another limitation was the demanding nature of the trial, which required children to maintain their attention and motivation over 5 consecutive days. This may have been difficult, particularly for our sample with co-occurring attention, learning and anxiety disorders, and may have contributed to performance variability. Co-morbidities and the fact that children with DCD are a heterogeneous group who display many different types of motor skill deficits, constitutes a significant challenge for future trials.

## Conclusion

Children with DCD demonstrated motor learning as measured by the PPT with retention of acquired skill at 6-weeks. The addition of motor cortex tDCS during training did not enhance motor learning, as seen in other populations. Procedures were well-tolerated and appear safe. Before conclusions can be made regarding the efficacy of tDCS in DCD, additional carefully designed trials with reproducible results are required. Establishment of an optimal tDCS protocol in DCD is essential, including stimulation target and montage.

## Data Availability Statement

The raw data supporting the conclusions of this article will be made available by the authors, without undue reservation.

## Ethics Statement

This study, involving human participants, was reviewed and approved by the University of Calgary Conjoint Health Research Ethics Board (Ethics ID: REB10-0183). Written informed consent to participate in this study was provided by the participants' legal guardian/next of kin. Written informed consent was obtained from the minor(s)' legal guardian/next of kin for the publication of any potentially identifiable images or data included in this article.

## Author Contributions

MG, AK, and DD conceptualized and designed the study. MG collected, analyzed and interpreted the data, and took the lead in writing the manuscript. BC assisted with the data analysis. DD and AK secured grant funding for this study, provided, administrative, technical, material support, and supervised the project. BC, AK, and DD provided critical feedback and helped to shape the final manuscript. All authors contributed to the article and approved the submitted version.

## Conflict of Interest

The authors declare that the research was conducted in the absence of any commercial or financial relationships that could be construed as a potential conflict of interest.
